# Tailoring Optical Performance of Polyvinyl Alcohol/Crystal Violet Band-Pass Filters via Solvent Features

**DOI:** 10.3390/polym16233288

**Published:** 2024-11-26

**Authors:** Raluca Marinica Albu, Iuliana Stoica, Simona Luminita Nica, Marius Soroceanu, Andreea Irina Barzic

**Affiliations:** “Petru Poni” Institute of Macromolecular Chemistry, 41A Grigore Ghica Voda Alley, 700487 Iasi, Romania; albu.raluca@icmpp.ro (R.M.A.); stoica_iuliana@icmpp.ro (I.S.); nica.simona@icmpp.ro (S.L.N.); soroceanu.marius@icmpp.ro (M.S.)

**Keywords:** polyvinyl alcohol, crystal violet, colorimetry, refractivity, transmittance, morphology, band-pass filter

## Abstract

Optical filters are essential components for a variety of applicative fields, such as communications, chemical analysis and optical signal processing. This article describes the preparation and characterization of a new optical filter made of polyvinyl alcohol and incremental amounts of crystal violet. By using distinct solvents (H_2_O, dimethyl sulfoxide (DMSO) and H_2_O_2_) to obtain the dyed polymer films, new insights were gained into the pathway that underlies the possibility of tailoring the material’s optical performance. The effect of the dye content on the sample’s main properties was inspected via UV–VIS spectroscopy analysis combined with colorimetry, refractometry and atomic force microscopy experiments. The results revealed that the colorimetric parameters are affected by the dye amount and are dramatically changed when the solvent used for film preparation is different. The rise in the refractive index upon polymer dyeing was due to the synergistic effect of the larger polarizability of the dye and the occurrence of hydrogen bonds among the system components. Spectral data evidenced that samples prepared in H_2_O and DMSO preserve the absorption characteristics of the added dye, whereas H_2_O_2_ acts as an oxidizing agent and enhances transparency. Also, for the first two solvents, multiple absorption edges were noted as a result of dye incorporation, which was responsible for the occurrence of new exciton-like states, hence the band gap reduction. The films processed in H_2_O were able to block radiations in the 506–633 nm range while allowing other wavelengths to pass with a transmittance above 90%. The samples attained in DMSO presented similar properties, with the difference that the domain of light attenuation was shifted towards higher wavelengths. Atomic force microscopy showed the dye’s effect on the level of surface roughness uniformity and morphology isotropy. The dyed polymer foils in non-oxidizing agents have suitable features for use as band-pass filters.

## 1. Introduction

Optical filters (OFs) represent a category of devices that are capable of transmitting radiations from specific intervals of wavelengths while blocking others [1]. In other words, OFs are components that are characterized by transmittance or reflectance, which varies with frequency or wavelength. Other OFs exhibit a reliance on polarization or spatial distribution, or they offer a uniform degree of attenuation [2]. These optical elements play an essential role in numerous technical applications, ranging from displays, chemical analysis, optical communications and sensing systems to everyday photography [3,4,5]. As the aforementioned technologies keep advancing, the significance and complexity of OFs are expected to increase.

A particular type of OF is band-pass filters (BPFs), which can allow particular frequencies from the spectrum to pass (rendering a band of high transmission) and reject (attenuate) frequencies outside the selected spectral zone. This sort of OF can be described by cut-on and cut-off slopes depicting the transition between strong rejection and elevated transmission. BPFs can be divided into two categories, which are absorption filters and optical interference filters [1,6]. The first type works based on the absorption of unwanted wavelengths, whereas they selectively transmit radiations of exact wavelengths. They are made through the insertion of organic dyes, chemical pigments or even rare earth transition metals in glass/plastic materials. The BPF efficiency of radiation transmission in a particular spectral range is dependent on factors like the content of dye or pigment in the OF, its thickness and also the optical quality of the glass or polymer employed in the manufacturing process. Material quality is crucial since it should be characterized by uniformity and density across the OF’s overall surface [7]. Absorption BPFs are used in practical cases where the transmission of precise frequencies and isolation of broad bands are required, namely in camera lenses for photography devices, components for fluorescence microscopes, light indicators for traffic, etc. The second type of BPF relies on leveraging reflection or destructive interference to transmit certain wavelengths and reject others [1]. They are made of multilayered coatings applied to optical-grade glass or polymers. When radiations reach this sort of BPF, the component layers transmit certain wavelengths, and at the same time, they reflect nonessential ones. The spectral range that can be transmitted or reflected is impacted by the features of the layered surface and the number of layers. The filtering precision of interference BPFs arises from the reflective cavities produced among the filter layers. In spite of their remarkable wavelength selectivity, interference BPFs are not as durable as absorption BPFs and are affected by the radiation incidence angle since the phase thickness and equivalent admittance of the filter’s sheets are influenced by this factor [6]. Over the past few years, several studies [8,9,10] have been devoted to the development of BPFs for understanding the factors that are able to drive their optical behavior towards better functionality. To date, it has been found that a BPF must have certain features, such as an average transmission above 80% (which allows for optimal radiation throughput), steep slopes (which enable a good isolation of the desired wavelengths), adequate blocking/strong optical density (effectively reducing undesired radiation leakage) and a tight control of the refractive index (uniform velocity of radiations through the OF). BPFs can be fabricated to display a broad range of performance characteristics, from the basic single-cavity [11] and unblocked Fabry–Perot [12] configuration to intricate designs, such as multi-mode [13], fully blocked [14] and all-dielectric designs [15].

Aside from the envisioned designs of BPFs, their efficiency resides in the properties of the constituting materials. Polymers seem to be advantageous for the fabrication of BPFs due to their excellent optical clarity, flexibility, light weight and reduced costs [16,17,18]. El-Gamal et al. [19] used poly(methyl methacrylate) (PMMA) and brilliant cresyl blue to prepare BPFs and found that a cut-off transmission takes place in the domains of 200–366 nm and 560–660 nm for 2 wt% dye. Also, they observed that the presence of dye molecules generates several absorption edges while increasing the Urbach energy and lowering the band gap energy. Hwang and Kim [20] made near-infrared (NIR) BPFs for CMOS image sensors by utilizing a NIR-absorbing dye (Epolight 1178), transforming Ag nanodisks into a cyclo-olefin transparent polymer. The prepared NIR BPF permits NIR radiations to pass in a low percentage (~6%) in the range of 700–800 nm, while the transmittance is about 84% in the interval of 400–680 nm.

On the other hand, there are papers focused on exploiting polyvinyl alcohol (PVA)/dye systems that have optical properties with potential utilization as absorption OFs. This polymer is known to be largely transparent in the visible domain and is easily processable by stretching [21]. Das et al. [22] inserted ZnS:Cu quantum dots in PVA to achieve low-pass OFs. Their experiments showed that the doped samples present an absorption peak at 301 nm, while photoluminescence spectra contain an emission peak at 400 nm. Bisen et al. [23] mixed PVA and PMMA and incorporated coumarin dye and observed that PMMA’s optical behavior was more impacted by the dye molecules than PVA, whereas the band gap energy of the pristine polymers was reduced upon doping. Rejini et al. [24] prepared xanthene/PVA systems and measured their absorption characteristics over 200–650 nm. They remarked that the samples display a narrow band pass, a narrow attenuation band and also a green laser beam-blocking behavior. Ali et al. [25] incorporated methyl violet-6B in PVA and spectral experiments, evidencing that the dye molecules influence absorption. The latter was enhanced in the 200–350 nm zone, but full radiation blocking took place in the interval of 500–625 nm, as required for making band-stop OFs. Durgesh et al. [26] employed Ag-Ni core–shell particles and introduced them in PVA to obtain UV-region BPFs. Analyzing the absorption characteristics, they noted that the samples present a sharp band with a maximum of about 320 nm, which is adequate for the pursued scope. El-Bashir et al. [27] synthesized rose bengal/PVA composites and demonstrated that, at 10 wt% dye, the films exhibit great optical cut-off features in the domain of 600–1400 nm with an elevated transmission value of 84%, as demanded for long-pass OFs. Zelinschi et al. [28] associated Congo Red with PVA and noticed a modification of morphology and optical characteristics in regard to the unfilled polymer. Also, by applying uniaxial drawing, they proved that the dichroic ratio and birefringence are enhanced at larger dye contents. Since the dispersion of the dichroic ratio has been lowered, their samples are good candidates as polarizing filters. Dorohoi et al. [29] inserted in PVA some pyridinium ylids and subjected the dyed films to stretching. They observed augmentation of the dichroic absorption as the induced ordering level is higher, so these foils have applicative potential as polarizing OFs. In another work [30], PVA was mixed with three different azo-derivative dyes and then the systems were stretched, emphasizing that the chemical structure of the dye had a powerful impact on the dichroism, and this is useful for making interference filters. Ghoshal et al. [31] dyed PVA with methylene blue and attained low transmittance in the UV region upon doping and a very small absorption above 705 nm, accompanied by a reduction in the band gap energy. Therefore, their materials can be regarded as candidates for power laser cut-off filters in the UV–VIS spectral zone. In a recent study, Obeed et al. [32] doped PVA with crystal violet (CV) and studied the influence of dye content in PVA solutions attained in deionized water and the film thickness on the absorption coefficient, band gap and refractive index, but no discussions on the applicative potential were made.

As mentioned above, there are few studies on the use of dye-doped PVA films as BPFs, and no work has been published on the relation between the solvent type (used for making the OF) and the resulting optical properties of the PVA/CV films. The current work describes the preparation of novel BPFs based on PVA and CV, which were solubilized in three distinct solvents, namely water (H_2_O), dimethyl sulfoxide (DMSO) and hydrogen peroxide (H_2_O_2_). The changes in the sample properties were evidenced by characterizations via colorimetry, refractometry, UV–VIS spectrometry and atomic force microscopy (AFM). The data acquired from these techniques will enable the extraction of new insights on the possibility of tailoring the performance of the obtained PVA-based BPF.

## 2. Materials and Methods

### 2.1. Materials and Sample Preparation

**Scheme 1:**
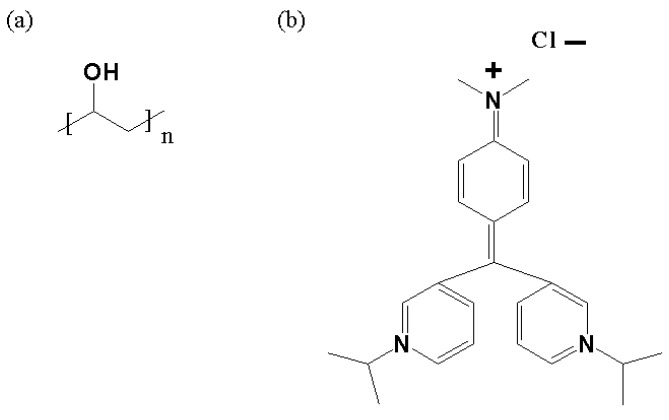
The chemical structures of (**a**) PVA structural unit and (**b**) CV molecule.

The polyvinyl alcohol (PVA) powder was acquired from Sigma-Aldrich (St. Louis, MO, USA), and according to the provider, it has a molecular weight of ~89,000 g/mol and is 99% hydrolyzed. The crystal violet (CV) was purchased from the Vitalia K SRL (Ploiesti, Romania) producer. DMSO (ACS reagent, ≥99.9%) was purchased from the Sigma-Aldrich (St. Louis, MO, USA) and H_2_O_2_ (solution 3%) was taken from the Vitalia K SRL (Ploiesti, Romania). The chemical structures of the investigated compounds are illustrated in Scheme 1.

**Scheme 2:**
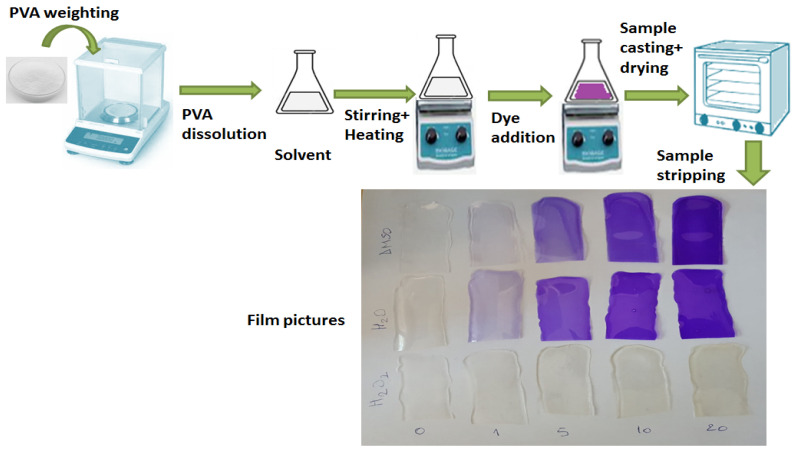
The scheme of preparation of the PVA and PVA/CV films from the solutions in the selected solvents and pictures of the samples.

The PVA/CV samples were attained by applying the protocol illustrated in Scheme 2: (a) 1 g of polymer was solvent in 8 mL of solvent; (b) all these prepared solutions were homogenized by heating at 90 °C and magnetic stirring for 2 h; (c) in each PVA solution variable amounts of CV were added (1 to 20 UI, where 100 UI = 1 mL) and the systems were subjected to continuous stirring for 2 h; (d) the PVA and PVA/CV solutions were cast on glass slides and left to dry in an oven (Binder Gmbh, Tuttlingen, Germany) at temperatures varying between 50–100 °C during 96 h; and (e) the dried films were stripped from the supports and further analyzed.

The chosen solvents for sample preparation were H_2_O, DMSO, and H_2_O_2_. According to Table 1, these solvents differ in terms of the refractive index and Hansen solubility parameters (HSP): the dispersion component of the HSP (δd), the polar component of the HSP (δp) and the hydrogen bonding component of the HSP (δh)—which were taken from the literature [33,34,35]. Also, the polarizability, refractivity and dipolar moment were calculated from simulations with the demo version of HyperChem. The optimized structures were attained using Molecular Mechanics (Amber force field) and the Polak–Ribiere conjugated gradient until the energy gradient was below 0.001 kcal/mol Å (maximum cycles 600, screen refresh period of 50 cycles). These geometry optimizations allowed us to obtain the most stable conformation with a minimum of energy, and from this state, we obtained the solvent physical parameters (Table 1) on the basis of the methods available in this software package. Once minimization was finished, the data obtained from the Quantitative Structure–Activity Relationship (QSAR) module included the predicted values for polarizability and refractivity. Moreover, based on a single-point energy calculation along with a semi-empirical approach, the dipole moment for the molecular structure of used solvents was acquired.

The film thickness was recorded on a digital micrometer, and it was noted to vary between 0.08–0.16 mm. The free-standing polymer films with or without dye were subjected to various analyses to check their performance.

### 2.2. Characterization Methods

Colorimetry analyses of the pristine and dye-containing specimens were undertaken on the CL-70F system (Konika Minolta, Tokyo, Japan).

Refractometry experiments were conducted on a refractometer (Anton Paar GmbH, Ashland, VA, USA) under ambient conditions at three wavelengths: 486, 589 and 656 nm.

UV–VIS spectrometry was carried out on the SPECORD 210 PLUS (Analytik Jena, Jena, Germany) for the pure polymer and doped polymer free-standing films.

Atomic force microscopy (AFM) data were achieved on an NTEGRA multifunctionalscanning probe microscope (NT-MDT Spectrum Instruments, Moscow, Russia).

## 3. Results and Discussion

This part of the paper describes the main colorimetric, optical, spectral and morphological characteristics of the PVA and PVA/CV samples in relation to their applicative potential as BPFs, emphasizing the role of the solvent quality in tailoring the material performance.

### 3.1. Colorimetry Analysis

**Figure 1:**
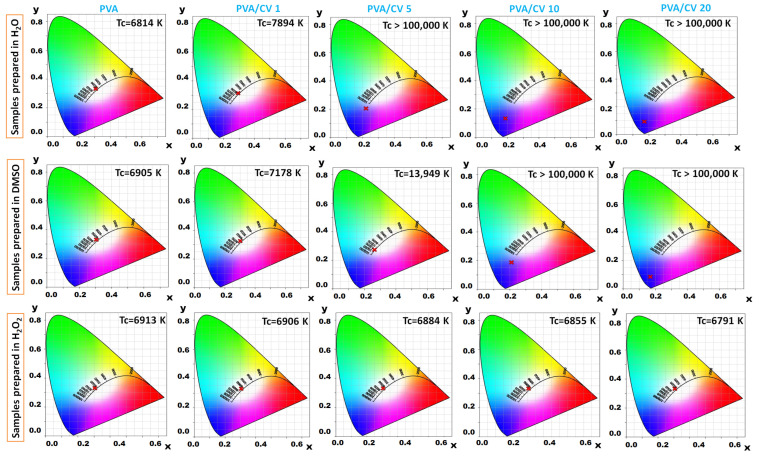
The chromaticity diagrams (CIE 1931) recorded for PVA and PVA/CV films prepared from the corresponding solutions in the selected three solvents.

The presence of the CV molecules in the PVA matrix produces relevant changes in the film’s appearance. As noted in Scheme 2, the color of the films differs as a function of the dye content and is significantly affected by the nature of the chosen solvent. The color-related features were attained by placing the PVA-based films on an illuminance meter that incorporates a spectrometer and a CMOS image sensor. All experiments were done under ambient LED light exposure. Figure 1 illustrates the chromaticity diagrams (CIE 1931) registered for the PVA and PVA/CV foils.

It was found that the CIE color spaces are influenced by the content of the inserted CV dye in the polymer and also by the type of solvent used for the preparation of the specimens. The experiments enabled an evaluation of the correlated color temperature (Tc) that expresses the color appearance of the radiation produced by a light source and depicts its color in relation to that of a reference [36]. The values of Tc are included in Figure 1 for both neat and dyed polymer films. The pristine PVA foils present small differences in Tc magnitude, which reflect the role of the used solvent. Upon gradual addition of the dye molecules in the transparent macromolecular matrix, Tc exhibited a different variation tendency; namely, it was higher for systems in H_2_O and DMSO, while it was lower for systems in H_2_O_2_. An additional description of the color characteristics involved analysis of the CIE color space, which refers to the trichromatic coefficients (x, y and z). They are further linked to the normalized values of the tristimulus parameters (X, Y and Z) [37]. These data, collected from the performed experiments, are summarized in Table 2. The recorded numerical values are computed by the device software by accounting for the illuminants’ spectral power distribution.

Regardless of the solvent features, the magnitude of the tristimulus parameters decreased when the CV amount in the matrix ranged from 1 to 20 UI in comparison to pure PVA. For understanding the evolution of the chromatic characteristics when both the solvent and sample composition were changed, the CIELAB coordinates (L*, a* and b*) were evaluated using the Relations (1)–(3):

L* = 116 ∙ (Y/Yn)^1/3^ − 16, (1)

a* = 500 ∙ [(X/Xn)^1/3^ − (Y/Yn)^1/3^], (2)

b* = 200 ∙ [(Y/Yn)^1/3^ − (Z/Zn)^1/3^], (3)

where L* designates the lightness parameter (0 value for black, 100 value for white), a* designates the red/green parameter and b* designates the yellow/blue parameter, while Xn, Yn and Zn designate the reference tristimulus data.

According to Table 2, the lightness parameter is almost invariable with the CV content for the samples prepared in H_2_O_2_, whereas, for those attained in DMSO and water, the magnitude of L* decreases due to the progressive inclusion of the dye molecules in PVA. Larger differences in the sample’s color were monitored using the a* and b* parameters. The neat PVA exhibits positive and close to 0 values of a*, which reveal a neutral hue. Additionally, the PVA/CV system in H_2_O_2_ has similar properties, owing to the solvent oxidizing action. Discoloration and photo-oxidation of the CV in the presence of H_2_O_2_ were previously reported [38]. For the foils prepared in H_2_O and DMSO, the incorporation of the dye led to an increase in the a* values, which means a shift towards the red hues. The negative values of the b* parameter correspond to the blue hue. This aspect is accentuated by the addition of CV in PVA in H_2_O and DMSO, and it is maintained even for the specimens processed in H_2_O_2_. The blue hue is more dominant for the systems prepared in H_2_O, which forms both donor and acceptor types of hydrogen bonds with polymer and dye. In the case of DMSO, the shade of blue slightly decreased since these bipolar aprotic molecules display powerful acceptor characteristics of hydrogen bonding, which reduces the overall system’s polarity [39].

**Figure 2:**
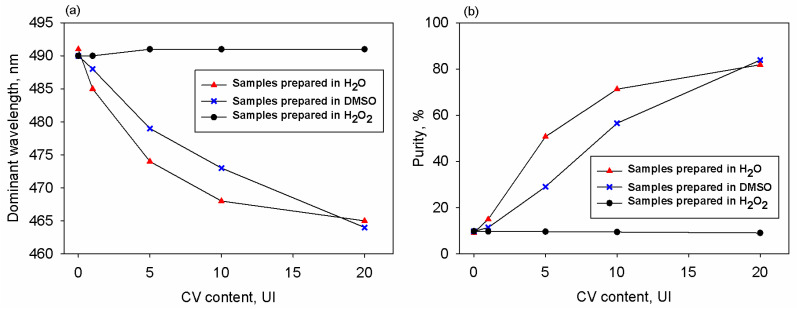
(**a**) Dominant wavelength and (**b**) excitation purity versus CV content for the PVA and PVA/CV films prepared from the corresponding solutions in selected three solvents.

Other relevant colorimetric parameters are the dominant wavelength and excitation purity, which are plotted in Figure 2 as a function of the CV content. The dye incorporation in PVA reduced the magnitude of the dominant wavelength for the specimens in H_2_O and DMSO. However, when H_2_O_2_ was used, the dominant wavelength of the dyed films remained almost unchanged, as seen in Figure 2a. Moreover, any color with a dominating wavelength is depicted by the excitation purity. The latter is defined as the ratio of the distances in the chromaticity diagram that shows the extent of the color shifting from the achromatic color toward the spectrum color. According to Figure 2b, the excitation purity of the radiation (passing through the dyed samples) increased to a larger extent for the specimens prepared in H_2_O and DMSO and was independent of composition for the samples in H_2_O_2_.

### 3.2. Refractive Index Dispersion and Optical Conductivity

**Figure 3:**
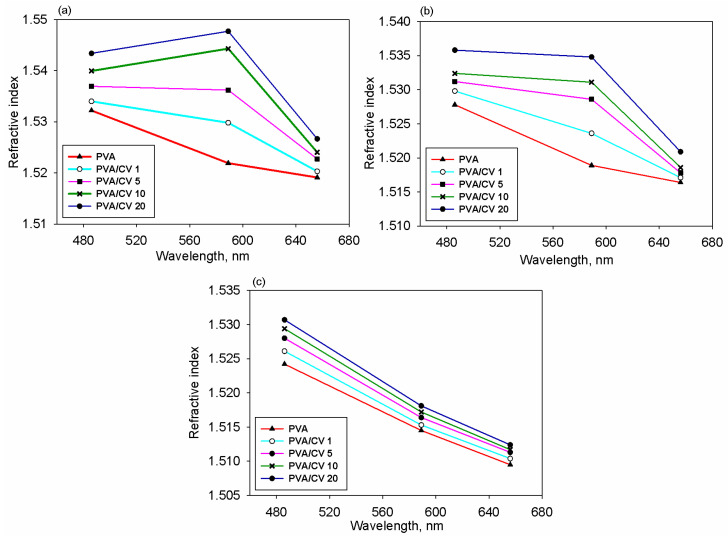
The influence of CV content on the refractive index dispersion for the PVA and PVA/CV films prepared in (**a**) H_2_O, (**b**) DMSO and (**c**) H_2_O_2_.

The refractive index (n) is widely utilized to extract information on the radiation propagation speed through an optical medium. In the case of studied materials, refractometry experiments were conducted to clarify the effect of the CV content on the optical properties of the PVA films. The registered data, illustrated in Figure 3, indicate that the dispersion curves are shifted upwards as the dye content is higher. The result accounted for the larger polarity of the CV molecules and their ability to interact via hydrogen bonding with the PVA. Literature [40,41] supports this by showing that there is a synergism between such interactions and high polarizability, which determines the increase in the refractive index.

In the spectral domain allowed by the refractometer, the variation in n with the light wavelength changes by the solvent type and dye presence/absence. For the non-colored PVA films, the refractive index becomes lower as the wavelength increases (normal dispersion). The incorporation of the CV (which displays the maximum absorption around 580 nm and two narrower peaks under 400 nm [42]) enhances the absorption of the dyed polymer foils. This is noticed for the samples processed in H_2_O and DMSO and not for the films achieved in H_2_O_2_ (as shown in the next section of the article). The dye-induced absorption of the PVA films is reflected in the refractive index changes with photon energy. The CV absorption features determine the increase in the refractive index with the wavelength. This is why the samples prepared in H_2_O and DMSO display an increase in the refractive index around 589 nm (anomalous dispersion), as seen in Figure 3. For the specimens attained in H_2_O_2_, the values of the refractive index decreased at larger wavelengths (normal dispersion). Similar behavior was previously reported for PVA/CV films obtained in deionized water [32].

On the other hand, the refractive index varies as a function of the solvent features (listed in Table 1). The small H_2_O molecules easily penetrate the PVA macromolecular coils. This solvent is capable of forming both donor and acceptor hydrogen bonds with the system components. Literature [43] indicates that during H_2_O removal from PVA, a bigger crystallization rate is favored since the mobility of the polymer chains is improved by the lessening of hydration hydrogen bonds formed among the chains. Hence, the drying process reduces the free volume and strengthens the PVA intermolecular hydrogen bonds [44]. It is well known that the increase in the polymer crystallinity is related to denser chain packing [45], which, in turn, generates an increase in the refractive index [46]. Based on this, one may explain the higher refractivity of the PVA foils made from aqueous solutions. Another report [44] reveals that H_2_O absorption determines the faster complexation speed of the PVA in the presence of a dye, which limits the chain segments’ movement due to the appearance of an intermolecular ordered structure. Moreover, the addition of polarizable CV molecules in PVA, combined with strong polymer–dye interactions (i.e., hydrogen bonding), produces a synergism that enhances the refractive index, as supported by literature data [41]. When the film samples are prepared in the DMSO solutions, several aspects should be accounted. First, such a bipolar aprotic compound enables the formation of hydrogen bonds of solely acceptor characteristics, which are less polar than those having donor characteristics [39]. Secondly, compared to water, the DMSO molecules have a larger size, so their diffusion among the polymer chains is slowed down. It was reported that the occurrence of hydrogen bonds between this solvent and PVA determines the restriction of the PVA crystalline zones growth, leading to smaller dimension of the crystallites [47]. Hence, after DMSO removal, these aspects will lead to fewer molecular dipoles (smaller polarity) compared to the previous case, explaining the slight reduction in the refractive index. Also, the incorporation of CV in these systems generates an increase in the n parameter. In the case of the specimens prepared in H_2_O_2_ solutions, the literature acknowledges the oxidizing activity of this solvent in the presence of either PVA [48] or CV [38]. A previous report [49] demonstrates that this solvent produces a slight degradation of the PVA chains, especially under UV exposure. Also, the H_2_O_2_ solvent interaction with CV triggers the decolorization and photodecomposition of this dye, leading to a colorless and less conjugated substance (smaller polarizability) [38]. Based on these aspects, the lower refractive values of these samples were explained.

Optical conductivity (σ_o_) and electrical conductivity (σ_e_) of the samples are further evaluated using the Relations (4) and (5) [50]:

σ_o_ = α∙n∙c/4π, (4)

σ_e_ = 2λ ∙ σ_o_/α, (5)

where α is the absorption coefficient determined from the spectrometry data, c is the speed of light and λ is the wavelength.

**Figure 4:**
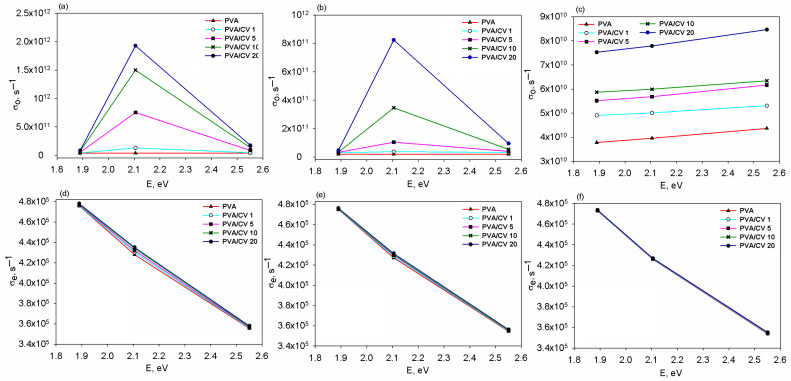
Optical conductivity for the PVA and PVA/CV films prepared in (**a**) H_2_O, (**b**) DMSO and (**c**) H_2_O_2_, and electrical conductivity for the samples prepared in (**d**) H_2_O, (**e**) DMSO and (**f**) H_2_O_2_.

The dependence of both σ_o_ and σ_e_ on the photon energy for the investigated samples is plotted in Figure 4.

Given the increased absorption of the films generated by the presence of the dye molecules, the optical conductivity increases in the entire photon energy interval in comparison with the pristine polymer. The largest increase of σ_o_ was observed for the samples prepared in H_2_O (Figure 4a) and DMSO (Figure 4b), while the lowest variation was remarked for the samples processed in H_2_O_2_ (Figure 4c). Hence, the foils processed in the first two solvents displayed suitable features for optoelectronic applications, owing to their enhanced optical conductivity, which reveals a good photoresponsive behavior. The results of the σ_o_ parameter show a link between the refractivity and absorption characteristics of the samples. The upward variation tendency of σ_o_ is ascribed to the generation of the localized energy levels in the sample’s structure. This is produced by the incremental addition of CV that renders a more compact distribution of the localized phases. The plots depicted in Figure 4d,e reveal how σ_e_ ranges at variable photon energies in the dyed polymer foils. It was found that the electrical conductivity is diminished at higher energies in both the pristine and doped PVA films. For the films made in H_2_O_2_, the insertion of CV in PVA produces less significant variations in the conduction features. Conversely, for the samples made in the H_2_O and DMSO solutions, a larger CV content leads to slightly higher values of the σ_e_ parameter due to the increase in charge carriers. Thus, these dyed films are good candidates for optoelectronic and photoelectric devices.

### 3.3. Transmittance and Band Gap Energy

**Figure 5:**
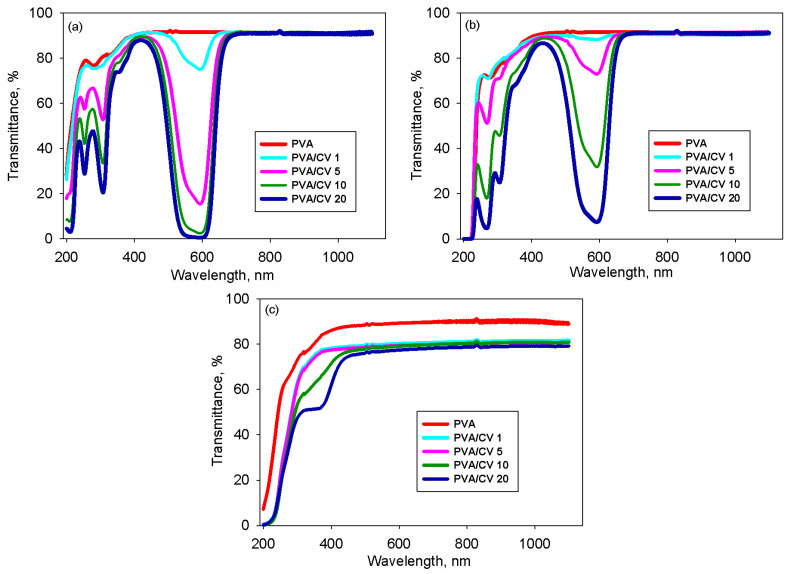
The transmittance versus wavelength for PVA and PVA/CV films prepared in solutions of (**a**) H_2_O, (**b**) DMSO and (**c**) H_2_O_2_.

The UV–VIS-NIR spectra (registered for the PVA/CV films) are illustrated in Figure 5a–c. It is largely known that PVA is a highly transparent material in the visible domain [26,31]. Differences in the transmittance magnitude of the studied pristine PVA foils are caused by the type of solvent. These can be discerned up to 430 nm for the systems attained in H_2_O and DMSO; afterward, the level of light transmission is similar, while the transmittance values of the samples in H_2_O_2_ are smaller over the entire interval of wavelengths.

The addition of CV in PVA produces the modification of the spectra shape as a result of the dye spectral features in the selected solvents. Literature [42] reveals that the aqueous solutions of CV display a wide absorption band, with the maximum being at 580 nm and two small peaks below 400 nm. Therefore, PVA/CV films prepared in H_2_O exhibit typical spectral characteristics for electronic excitations within the CV molecules, which cause noticeable absorption bands. Their intensity is higher as the CV concentration in the films is enhanced. Hence, the PVA film containing the highest CV content (20 UI) presents a sharp drop in the transmission values in the 506–633 nm range, while the other wavelengths pass with a transmittance beyond 90%. Consequently, the PVA/CV 20 foil leads to the best attenuation of these radiations, while the others are transmitted, except for those at 251 and 303 nm. For the colored polymer films achieved in DMSO, similar spectral zones are observed, with the difference that the absorption bands are easily shifted towards bigger wavelengths; namely, small peaks are found at 263 nm and 308 nm, while the main absorption band is slightly narrower (522–625 nm). This is in agreement with literature data [51], which prove that bipolar aprotic molecules, like DMSO, shifted the absorption spectrum of CV towards the long-wave domain. Oppositely, the spectra of the PVA/CV samples attained in H_2_O_2_ lack the aforementioned bands, which derive from the dye spectral features. This is due to the fact that this solvent acts as a powerful oxidizing agent, which produces CV photodecomposition and decolorization in the presence/absence of optical radiations and finally leads to the colorless leuco crystal violet [38]. Therefore, the PVA/CV films prepared in H_2_O_2_ have high transparencies starting at 400 nm. Also, as the dye quantity is larger, the magnitude of the transmission decreases. The films containing 10 and 20 UI of CV present a small shoulder below 400 nm, which might reflect a small absorption of a higher amount of products, which resulted in dye photodecomposition. Analysis of the UV–VIS spectra evidenced that the PVA/CV 20 film prepared in aqueous solutions display a higher ability to block radiation on a larger wavelength interval comparatively to the other two systems made in DMSO or H_2_O. Hence, it appears that the samples prepared in H_2_O have the best optical performance for BPF applications.

Further information on the absorption caused by the CV molecules inserted in the PVA transparent matrix was acquired by examination of the absorption coefficient (α) variation with the photon energy (E). For the investigated pristine and dyed PVA foils, α was determined using Equation (6):

(6) $$\alpha =\frac{1}{t_{0}}ln\left(\frac{1}{T}\right),$$

where t_0_ denotes the film thickness, and T denotes the transmittance.

**Figure 6:**
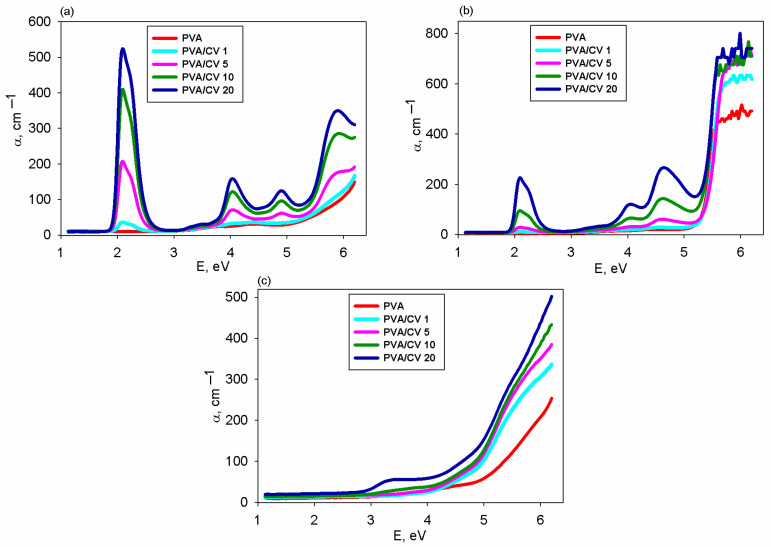
The plots of absorption coefficients against photon energy for PVA and PVA/CV films prepared in solutions of (**a**) H_2_O, (**b**) DMSO and (**c**) H_2_O_2_.

The absorption coefficient dependence on the incident photon energy is plotted in Figure 6 for all the examined pristine/colored PVA films. It was found that the manner in which monochromatic radiations penetrate the PVA-based samples, prior to their absorption, changed with both the dye content and solvent features. The data displayed in Figure 6 evidenced that the gradual incorporation of CV in the transparent PVA medium is responsible for the larger values of α. The films prepared in H_2_O and DMSO are characterized by increased α values. Also, the α = α(E) plots shown in Figure 6 present several absorption edges below 5.3 eV, which are better observed for the samples made in H_2_O rather than those attained in DMSO. The PVA and PVA/CV samples processed in H_2_O_2_ have lower α values and do not display multiple absorption edges. This might be caused by the fact that CV dye was photo-oxidized by this solvent [38].

The theory of band-to-band absorption in semiconductors was extended to materials having a lower structural order and it describes the types of optical transitions, which render the absorption edge and also the direct and indirect transitions in the k-space [52,53,54,55]. In such situations, an incident photon can excite an electron, which starts traveling from the valence band (VB) towards the conduction band (CB) and consequently exceeds the energetic barrier of the band gap energy (E_g_). The energy corresponding to the point where the absorption edge suddenly moves upward designates the electronic E_g_. Since there is always a direct E_g_ when the CB and VB extremes are found in the same point in k-space, the interband transition arises without the wave vector modification. In the opposite situation, the interaction with the lattice must occur to allow the transition between the bands. A phonon’s involvement in the optical transitions might lead to variations in the electron wave vector. Such transitions aided by phonons are typical of indirect E_g_ [52]. Because a phonon is implicated in indirect interband transitions, the absorption edge’s rising point deviates significantly in regard to the electronic E_g_. Aside from the nature of the optical transition, the density of the states of the analyzed material has a meaningful effect on the band-to-band absorption in the proximity of the band edge. A transition between the VB and CB is known to be either optically allowed or forbidden. By applying Tauc’s theory [54], one may acquire information on the E_g_ for the direct and indirect transitions by examining the α= α€ dependence in the vicinity of the band edge, according to Equation (7):

(7) $$\alpha E={\beta \left(E-E_{g}\right)}^{\gamma }$$

where β is a constant, and γ is a parameter for electronic transitions, which takes the value of 1/2 or 2 for the direct and indirect transitions [56].

**Figure 7:**
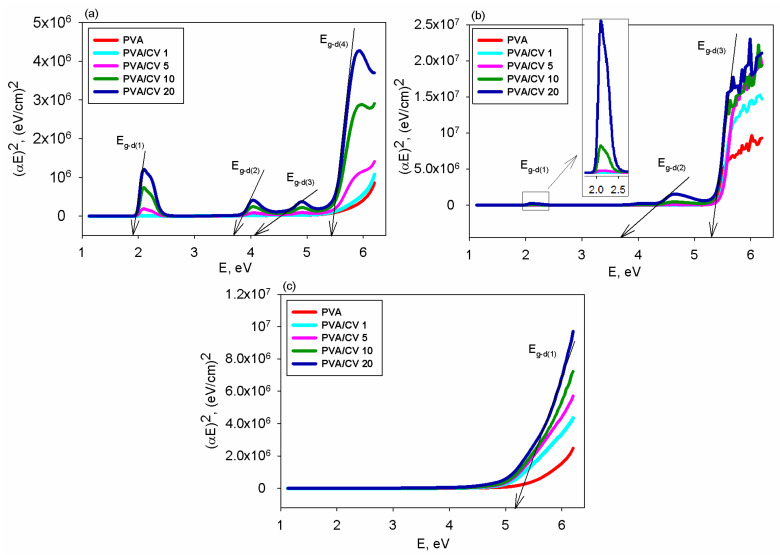
The dependence of (αE)^2^ on the photon energy for PVA and PVA/CV films prepared in solutions of (**a**) H_2_O, (**b**) DMSO and (**c**) H_2_O_2_.

**Figure 8:**
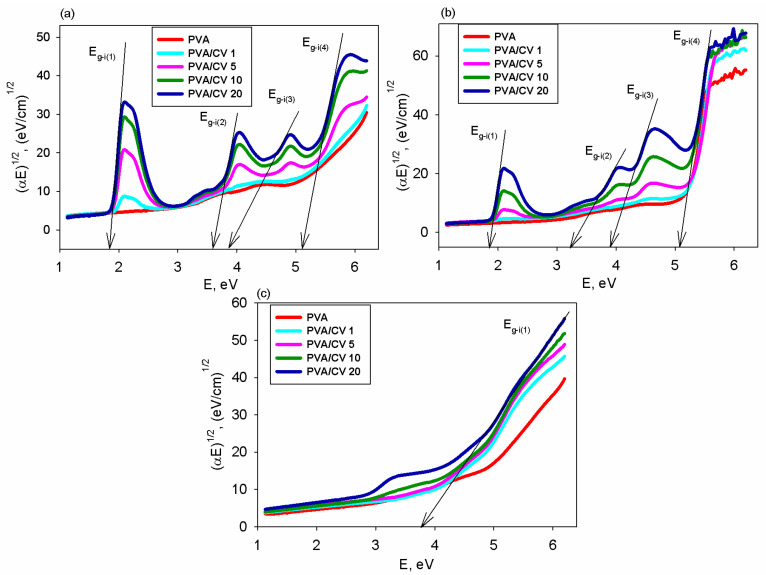
The dependence of (αE)^1/2^ on the photon energy for PVA and PVA/CV films prepared in solutions of (**a**) H_2_O, (**b**) DMSO and (**c**) H_2_O_2_.

Based on Tauc’s expression [54], it is facile to attain information on the direct and indirect band gaps by graphical plotting of the (α∙E)^2^ vs. E and (α∙E)^0.5^ vs. E, where the E_g_ values are achieved via extrapolation of the linear area to intercept the x-axis. These plots are shown in Figure 7 and Figure 8, whereas the extracted values for the direct band gap (E_g-d_) and indirect band gap (E_g-i_) of the PVA and PVA/CV films prepared from solutions in the selected solvents are listed in Table 3.

The data from Table 3 indicate that the magnitude of the E_g-d_ is higher than that of the E_g-i_ for the investigated films and that these values decrease as the dying level of the PVA is larger. Further insights were acquired from the analysis of the plot shapes, which are shown in Figure 7 and Figure 8. For all specimens, the absorption edges became more obvious with an incremental insertion of the CV in the transparent matrix. Figure 7a and Figure 8a reveal that the PVA films made in H_2_O present a bigger number of highly defined absorption edges, which are slightly shifted from the high energy for the neat polymer to smaller energy for the dyed samples. The polar and small H_2_O molecules have a high ability to interact via hydrogen bonding with the PVA and CV and are easily diffusing between the polymer chains, which favors the occurrence of additional intermolecular forces that stabilize the system. Upon drying, more sites of intermolecular hydrogen bonds among the PVA chains are formed, increasing the material’s density and overall polarity. These two aspects are related to the effective increase in the refractive index [41,46], which is known to be inversely proportional to the band gap energy, according to the theory of Moss [57]. By PVA coloration, the shifting of the absorption edge was ascribed to the absorption induced by CV, which generates novel exciton-like states. Also, this dye forms hydrogen bonds among the negative nitrogen atoms from the dye and hydroxyls from the PVA chains. Such bonding is known to display elevated polarity due to a big disparity in the atoms’ electronegativity [58,59]. The high absorption produced by the CV generates a sudden reduction in the optical band gap. This is supported by refractometry experiments, which indicate that dye introduction enhances the n parameter of the PVA/CV foils, and this implicitly corresponds to lower band gap energy, as noted in Table 3. The remarked shift of the sample’s band edge (upon gradual incorporation of the dye) concords well with the spectral deviation from the UV–VIS spectra. Figure 7b and Figure 8b show that the foils prepared in the DMSO solutions also present more absorption edges, but in this case, they are less defined. The aprotic nature and the larger molecule size of this solvent restrict the system’s polarity and area of the crystalline zones [39,47]. After solvent removal, fewer molecular dipoles are formed, as proved by the attained values of the refractive index, which are smaller than those registered for the foils made in H_2_O solutions. This is also sustained by the higher values of the band gap for the foils processed in DMSO (see Table 3). On the other hand, the oxidizing role of H_2_O_2_ on both PVA and CV is depicted in Figure 7c and Figure 8c, where a single absorption edge is observed. The corresponding band gap values are the highest in this case, compared to the PVA foils attained in the other two solvents. The addition of the dye, which photodecomposes in H_2_O_2_ into its less conjugated leuco counterpart, determines a slight decrease in the band gap (see Table 3). This is also shown by the moderate increase in the refractivity of these samples. All the above reveal that the solvent features are key factors in tailoring the band gap and the radiation-blocking ability of the prepared PVA/CV BPFs.

### 3.4. Morphological Analysis

**Figure 9:**
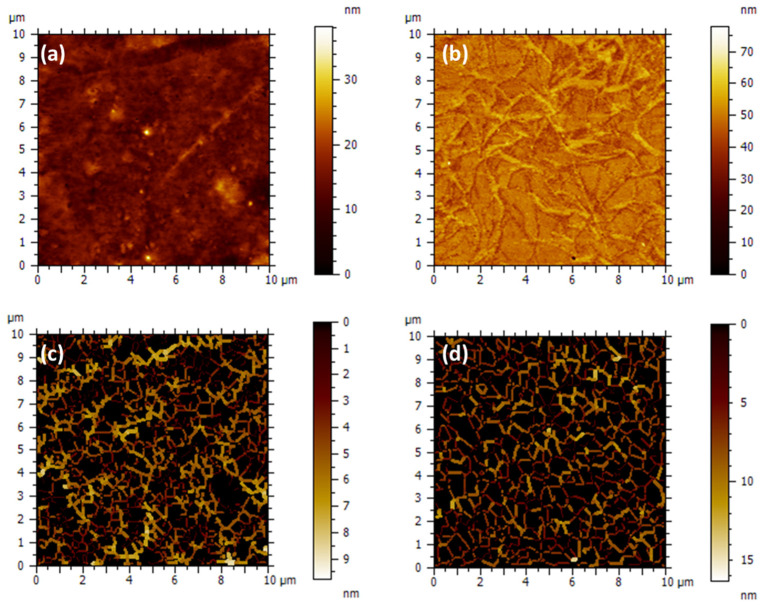
The height AFM images for (**a**) PVA and (**b**) PVA/CV films prepared in solutions of H_2_O and the corresponding images of the furrows analysis (**c**) and (**d**).

The dyed samples with the best optical performance were chosen for further investigation from the morphological point of view. Figure 9 presents the topographical AFM images of the PVA films derived from the aqueous solutions before (Figure 9a) and after the addition of 20 UI of CV dye (Figure 9b). At first, the surface of the neat PVA surface exhibits a morphology characterized by small globular formations typical of this polymer at this analytical level, which display uniformity without notable changes and result in a very low root mean square roughness (Sq), as indicated in Table 4. The PVA/CV/H_2_O sample reveals a totally different morphological aspect as a result of the incorporation of the dye into the system. For this sample, the Sq value increases by nearly 1.5 times; however, it remains quite small. When dealing with optical components, it is very important to analyze the material’s surface quality, specifically in terms of surface roughness. The latter is a paramount parameter that is deeply connected with the optical scattering phenomenon. Bennett and Porteus [60] elaborated a theory on the total integrated scatter (TIS), which was found to be affected by several parameters. They showed that the TIS factor is linked to the surface roughness; it is also proportional to reflectance, and additionally, it is inversely varying with the light wavelength. For instance, for a material having an Sq of 17 nm, the optical scattering is around 5%, which is considered to be the loosest tolerance for surface roughness. Thus, given the fact that the recorded values of the Sq parameter for the studied samples are under 3.9 nm, one may find that the optical scattering is lower than 5%, and this is suitable for the pursued application as OFs.

The layout of the recently developed structures is regular and lacks regions with distinct properties. This can be supported by examining the furrow analysis images in Figure 9c,d. Their appearance distinctly shows that, despite the dyed sample being slightly rougher, it exhibits superior uniformity of the roughness, and it misses any inhomogeneous areas.

**Figure 10:**
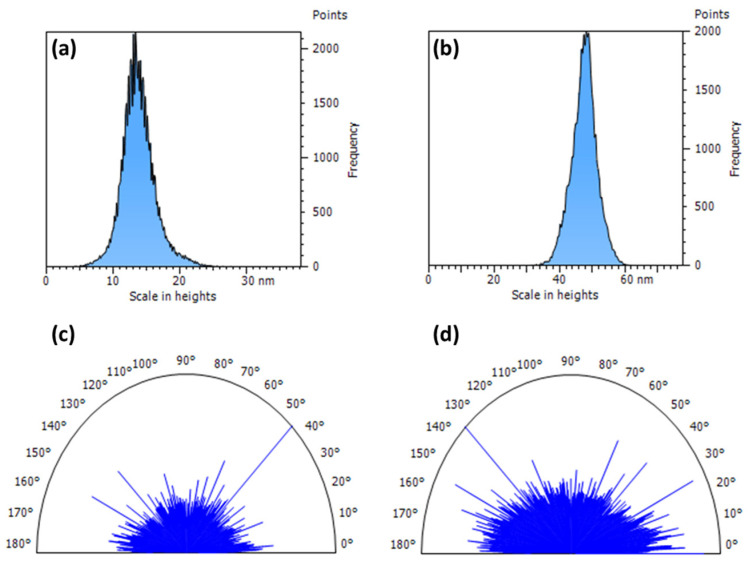
The height histograms and polar graphs of the texture direction, respectively, resulted from the AFM images for (**a**,**c**) PVA and (**b**,**d**) PVA/CV films prepared in solutions of water.

Concerning the height distributions depicted in Figure 10a,b, both samples exhibit a narrow profile, with the kurtosis values (Sku > 3) quantifying a certain degree of sharpness of the roughness feature. This is diminished for the specimen containing 20 UI of CV. Furthermore, the AFM scans enable the evaluation of several important parameters, namely the developed interfacial area ratio (Sdr), surface texture direction index (Stdi), and texture aspect ratio (Str). More details about them can be found in previous work [61], and the results are summarized in Table 4. The values of the Sdr parameter emphasize the higher complexity of the morphology for the sample containing the dye in regard to the pristine polymer foil. The level of morphology anisotropy can be quantified via Str and Stdi. The first parameter displays values that exceed 0.5 for both the pristine and CV-containing samples. This evidences a pronounced isotropy, which is slightly larger for the dyed PVA foil, so, for this specimen, the uniformity of the surface texture is higher. The Stdi values that are near unity revealed a lack of prevalent directions of the surface morphology, and this aspect was enhanced upon CV insertion in the PVA. All surface parameters related to the morphological features evidence that the dye presence in the transparent PVA is improving the level of morphology complexity and degree of isotropy so that light interacts uniformly with the studied dyed sample, which also has low roughness— an aspect that fulfills the criteria of a good BPF.

Another relevant aspect that must be accounted for is that the -OH groups from PVA are responsible for the phenomenon of water absorption from both aqueous solutions and the external environment [62]. Accounting for this, the water content of the prepared PVA foils is not the same. According to the literature [62], the moisture uptake of PVA might be avoided by the insertion of specific additives or by thermal crosslinking, which results in better mechanical properties. High-temperature annealing was found to remove water from the PVA and to render crystallization, while optical absorption in the visible range is greatly enhanced during heat treatment [62,63]. Moreover, at very high temperatures, the bulk, and especially superficial layers of the PVA films, were damaged so that the surface roughness was enhanced, owing to the occurrence of lower molecular weight products [64]. Therefore, excessive heating might remove the remaining water molecules from the PVA while generating undesired optical absorption and light scattering (because of the high roughness). Hence, future studies will focus on finding the best compromise between these factors for controlling the performance of the BPFs.

## 4. Conclusions

This work describes the preparation of distinct solvents of novel PVA/CV systems and their characterization in regard to their applicability as BPFs. New aspects were evidenced from the analysis of the solvent effects on the key properties of the samples. The colorimetric parameters revealed that the dye insertion in PVA has a dramatic impact on the resulting hues of the films, and the extent to which the color differences are noted depends on the solvent action on the system components. The occurrence of the hydrogen bonding of either donor/acceptor nature, combined with the higher polarity of the dye molecules, is responsible for increasing the refractive index of the colored PVA foils in H_2_O or DMSO. Conversely, the oxidizing action of H_2_O_2_ limits the increase in this optical parameter. Spectral studies showed that the absorption characteristic bands of the dye are maintained for the PVA foils processed in H_2_O or DMSO, while they disappear for the films made in H_2_O_2_. Such aspects determine the appearance of multiple absorption edges in the first two solvents and this is related to the exciton-like state formation and the band gap energy reduction, especially after dye incorporation. The samples attained in aqueous solutions were found to block the radiations in the 506–633 nm range and allowed the other ones to pass with a transmittance above 90%. DMSO has a similar effect, with the difference that it induces the shifting of the absorption band towards higher wavelengths. The AFM scans proved that the dye presence in PVA improved the roughness uniformity, morphology complexity and level of isotropy. These morphological properties are favorable for attaining proper interactions of light with the prepared PVA samples. The studied dyed polymer foils have the desired optical performance for BPF applications.

## Figures and Tables

**Table 1:** The main properties of the solvents used to obtain the PVA and PVA/CV solutions.

Solvent	Refractive Index *	Hansen Solubility Parameters, MPa^1/2^ **			Calculated Parameters from Simulations		
		δd	δp	δh	Polarizability, Å^3^	Refractivity, Å^3^	Dipole Moment, D
H_2_O	1.33	15.60	16.00	42.30	1.41	3.36	1.74
DMSO	1.48	18.40	16.40	10.20	5.01	20.56	4.48
H_2_O_2_	1.41	15.50	12.20	42.70	2.05	4.56	2.71

* Data from [33,34]; ** Data from [35].

**Table 2:** The values of tristimulus parameters and the resulting data for L*—lightness, a*—red/green color parameter and b*—yellow/blue parameter for all PVA-based systems.

System	CV Content, UI	X	Y	Z	L*	a*	b*
PVA/CV/H_2_O	0	4154.3712	4497.5076	4898.5273	96.6582	0.0918	−0.3890
	1	3730.7950	4000.1610	4980.4176	92.3423	1.5930	−8.9088
	5	1814.8195	1679.8231	4697.2185	65.1322	18.8166	−52.0406
	10	1219.2217	869.2384	4440.1880	49.1356	42.0053	−76.0544
	20	979.0900	526.7763	4185.5423	39.1207	62.4166	−89.6497
PVA/CV/DMSO	0	4218.4243	4566.2714	5035.3936	97.2295	0.1129	−1.2001
	1	4096.6821	4423.2108	5065.6116	96.0344	0.5219	−3.6526
	5	2750.4957	2853.5661	4827.5368	80.8058	6.0438	−26.7759
	10	1612.6946	1431.3740	4614.9630	60.9172	22.7616	−58.1811
	20	903.3637	453.7775	3975.3192	36.4469	65.9984	−91.1144
PVA/CV/H_2_O_2_	0	4188.7733	4533.7303	5005.2761	96.9599	0.1286	−1.2725
	1	4176.8625	4520.4744	4987.0259	96.8497	0.1415	−1.2240
	5	4214.3799	4563.5649	5015.9421	97.2071	0.0533	−0.9854
	10	4192.7908	4541.2413	4970.4316	97.0222	0.0155	−0.7091
	20	4179.0709	4530.4436	4908.6201	96.9326	−0.1301	−0.0495

**Table 3:** The values of the direct and indirect optical band gaps for PVA and PVA/CV films prepared in solutions of H_2_O, DMSO and H_2_O_2_.

System	CV Content, UI	E_g-d(1)_ (eV)	E_g-d(2)_ (eV)	E_g-d(3)_ (eV)	E_g-d(4)_ (eV)	E_g-i(1)_ (eV)	E_g-i(2)_ (eV)	E_g-i(3)_ (eV)	E_g-i(4)_ (eV)
PVA/CV/H_2_O	0	-	-	-	5.75	-	-	-	5.46
	1	-	-	-	5.64	-	-	-	5.34
	5	1.93	3.72	4.29	5.56	1.90	3.67	4.15	5.27
	10	1.88	3.75	4.18	5.47	1.88	3.62	4.02	5.16
	20	1.90	3.68	4.07	5.42	1.82	3.57	3.85	5.11
PVA/CV/DMSO	0	-	-	5.28	-	-	-	5.24	-
	1	-	-	5.32	-	-	-	5.20	-
	5	1.97	4.12	5.43	-	1.93	4.10	5.16	-
	10	1.93	3.84	5.49	-	1.89	3.39	5.10	-
	20	1.85	3.66	5.41	-	1.86	3.22	5.06	-
PVA/CV/H_2_O_2_	0	5.85	-	-	-	5.68	-	-	-
	1	5.56	-	-	-	5.51	-	-	-
	5	5.42	-	-	-	5.38	-	-	-
	10	5.28	-	-	-	5.25	-	-	-
	20	5.09	-	-	-	5.16	-	-	-

**Table 4:** The values of the 3D texture parameters calculated from the AFM images, height histograms and texture direction polar graphs for PVA and PVA/CV films prepared in solutions of water.

System	CV Content, UI	Sq, nm	Sdr %	Sku	Stdi	Str
PVA/CV/H_2_O	0	2.5	0.072	5.585	0.689	0.726
	20	3.9	0.583	4.287	0.812	0.750

## Data Availability

The dataset is available upon request from the authors.
